# Expression of Odontogenic Genes in Human Bone Marrow Mesenchymal Stem Cells

**Published:** 2013-07-02

**Authors:** Fatemeh Mashhadi Abbas, Hamed Sichani Fallahi, Ahad Khoshzaban, Nazanin Mahdavi, Seyedeh Sara Bagheri

**Affiliations:** 1 Department of Oral and Maxillofacial Pathology, School of Dentistry, Shahid Beheshti Medical Science University, Tehran, Iran; 2 School of Dentistry, Tehran University of Medical Sciences, Tehran, Iran

**Keywords:** *Pax9*, *DMP1*, Bone Marrow Stem Cells, Odontogenesis

## Abstract

**Objective:**

Tooth loss is a common problem and since current tooth replacement methods cannot counter balance with biological tooth structures, regenerating natural tooth structures has become an ideal goal. A challenging problem in tooth regeneration is to find a proper clinically feasible cell to seed.This study was designed to investigate the odontogenic potential of human bone marrow mesenchymal stem cells (HBMSCs) for seeding in tooth regeneration.

**Materials and Methods:**

In this experimental study, three pregnant Sprague Dawley (SD) rats were used at the eleventh embryonic day and rat fetuses were removed surgically using semilunar flap under general anesthesia. The primary mandible was cut using a stereomicroscope. The epithelial and mesenchymal components were separated and the dissected oral epithelium was cultured for 3 days. We used flow cytometry analysis to confirm presence of mesenchymal stem cells and not hematopoietic cells and to demonstrate the presence of oral epithelium. Bone marrow mesenchymal stem cells (BMSCs) and cultured oral epithelium were then co-cultured for 14 days. BMSCs cultured alone were used as controls. Expression of two odontogenic genes *Pax9* and *DMP1* was assessed using quantitative reverse transcription- polymerase chain reaction (RT-PCR).

**Results:**

Expression of two odontogenic genes, *Pax9* and *DMP1*, were detected in BMSCs co-cultured with oral epithelium but not in the control group.

**Conclusion:**

Expression of *Pax9* and *DMP1* by human BMSCs in the proximity of odontogenic epithelium indicates odontogenic potential of these cells.

## Introduction

It is well known that dental structures are derived from ectoderm and mesenchymal layers during embryogenesis which are almost destroyed after the formation of tooth structures (1). Therefore dental tissue exhibits a limited response to damages. Tooth loss will happen to most of people and may affect their life quality (2-5). Despite the technical improvements, the current tooth replacement methods and dental materials cannot counterbalance biological tooth structures (6). Stem cell-based tooth regeneration is a biological technique that aims to regenerate histological, morphological and functionally tooth like structures. Stem cells (SCs) are undifferentiated cells with multi-lineage differentiation and self-renewal capacity (7-9).

There are two types of stem cells according to their differentiation potential: embryonic stem cells (ESCs) (8) and somatic stem cells (also known as adult stem cells or mesenchymal stem cells) (10). Because of the limited usage of ESCs due to the ethical limitations (11), mesenchymal stem cells (MSCs) are applied as a more common source in tissue engineering (12). Post natal stem cells have been obtained from several sources such as periosteum, adipose tissue, skin, hair follicle, skeletal muscle, brain and bone tissue (13,14). In different studies six types of dental MSCs have been reported including: permanent dental pulp stem cells (DPSC) (15), stem cells from human exfoliated deciduous teeth (SHED) (16), human infantile dental stem cells (IDSC) (17, 18), periodontal ligament stem cells (PDLSC) (19), apical papilla stem cells (SCAPs) (20, 21) and dental follicle progenitors (22). Bone marrow is known as the best source of MSCs (17, 23). The origin of MSCs is different from follicle driven stem cells: they originate from mesoderm; however, the dental stem cells are driven from the neural crest (24). It has been demonstrated that the mouse BMSCs can differentiate into odontoblast and produce tooth-like structures in the proximity of embryonic dental epithelium (25-27). It has also been shown that produced tooth bud in the *in vitro* environment can be transferred into the adult mandible (26). BMSCs can also be extracted from the mandibular bone and it seems that the mandibular BMSCs have high osteogenic capacity (28). Nevertheless their count is much lower than iliac crest (29).

Dentin matrix protein1 (DMP1) is expressed in pulp and odontoblast cells during odontogenesis and facilitates mineral nucleus formation in special locations. It also prevents spontaneous calcium phosphate sedimentation in non-arbitrary sites (30). DMP1 is expressed before the expression of Dentin sialophosphoprotein (*DSPP*) gene (25) and regulates *DSPP* gene transcription, which indicates complete odontoblastic differentiation (31). Simultaneous expression of Paired box gene 9 (*Pax9*), *MSX1* and *Lhx7* is characteristic of dental ectomesenchymal tissue (32) and it has been suggested that these three genes can be used as an early event for the determination of odontogenic capacity (28). Expression of *Pax9* is mandatory for progression of tooth development; hence in its absence dental development will stop in bud stage. *DMP1* is not specific for dental tissue and is expressed by other hard tissue cells such as osteoblasts, osteocytes, ameloblasts and cementoblasts and functions by influencing the mineralization process of these tissues. It has also been demonstrated that *DMP1* is expressed in the brain tissue of mouse and cow (33). In the present study, we used human BMSCs since these cells are more available than dental stem cells and assessed the expression of odontogenic genes in these cells.

## Materials and Methods

### Isolation and culture of human bone marrow cells

Human bone marrow stem cells were obtained from Iran Transplant Productions Bank.

### Isolation and culture of rat oral epithelium

**Fig 1:**
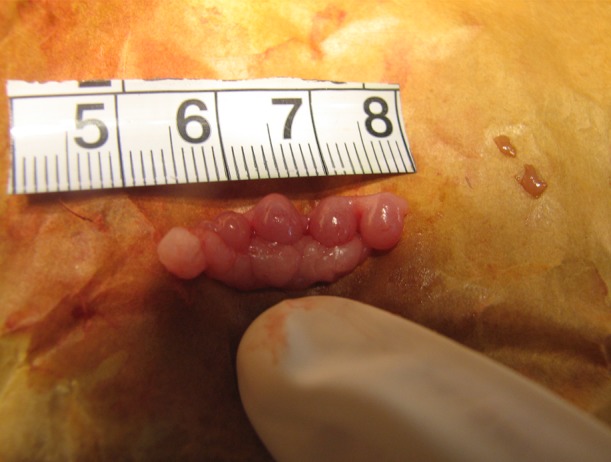
Rat fetuses were removed at the eleventh embryonic day.

Male and female sexually mature SD rats (3 females and one male) were placed in the same cage overnight. The following morning, if a plug was observed in the female rat’s vagina, then the fetal age was considered day 0. The pregnant SD rats were carefully weighed at both 0 and 11th embryonic days. Pregnant SD rats were used at the eleventh embryonic day (each pregnant rat has 8-10 fetuses) and rat fetuses were removed surgically under general anesthesia using Ketamin-Xylazine (1 ml/100 g/IP). Uterus was exposed using a semilunar flap (Fig 1). The primary mandible was cut and after the separation from the attached tissues was washed in PBS solvent for 5 minutes at room temperature, and cultured in medium (1:1 mix of DMEM, nutrient mixture ham’s F-12 medium, 1% penicillinstreptomycin) (Invitrogen, USA).

The mesenchymal and epithelial components were separated by incubating cells for 60 minutes in a solution (44 mM NaHCO_3_; 54 mM KCl; 110 mM NaCl; 0.9 mM NaH_2_PO_4_, 1mM sodium pyrovate, 42 mM phenol red pH=7.5, containing 1% penicillin-streptomycin, 1.4 mg/ml pronase and 0.1 mg/ml DNase, collagenase BB).

The epithelial cells were isolated using gentle movement and were cultured for 2 hours at 37˚C in a media (collection media with 5% FCS, 120 IU/ml insulin) unattached cells after washing were seeded in a count of 5×10^5^ cells /250 μl and incubated in 5% CO_2_, in humidity for 3 days. This passage was repeated for 4 times.

### Flow cytometry analysis

**Fig 2:**
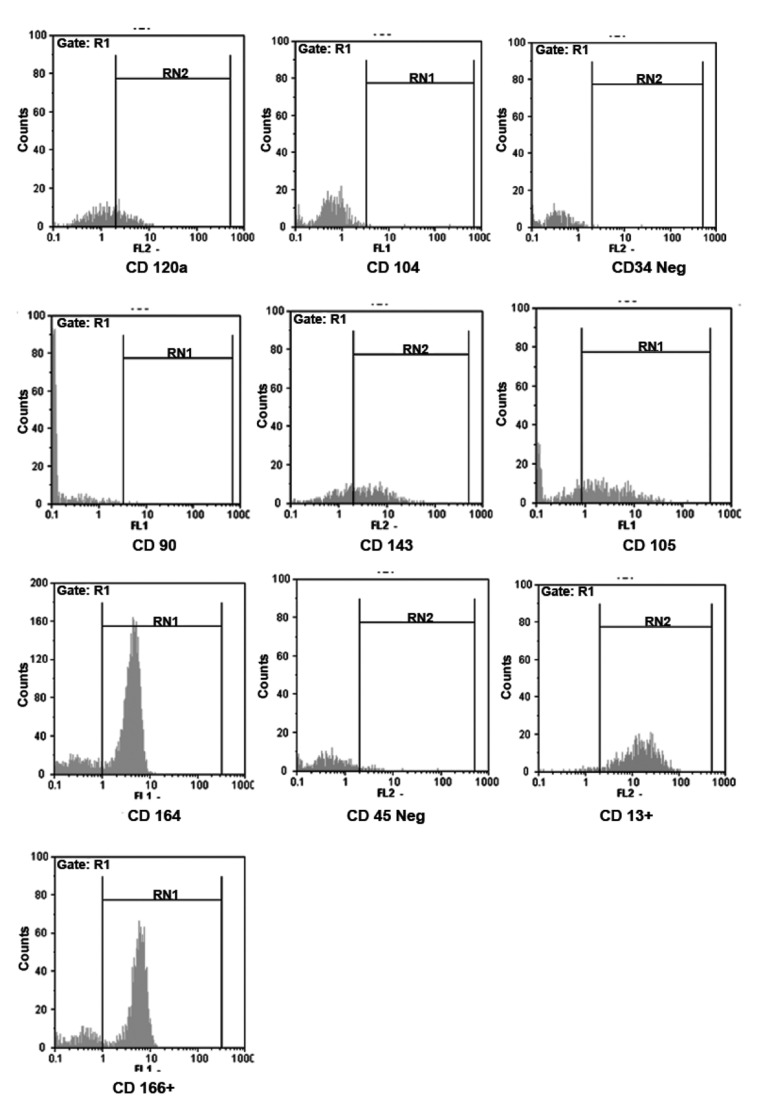
Flow cytometric analysis to confirm presence of oral epithelium (CD104, CD120, CD143 and CD164) and bone marrow mesenchymal stem cells (CD13, CD90, CD105, CD166, -34FITC and -45FITC).

After 4 times passage, cultured bone marrow cells were trypsinized and incubated with primary monoclonal antibodies against CD13, CD90, CD105, CD166, -34FITC and -45FITC to confirm presence of mesenchymal stem cells and not hematopoietic cells. Cultured embryonic epithelial cells were assessed for CD104, CD120a, CD143, and CD164 (Fig 2).

### Co-culture

The two single cell suspensions with the approximate count of 5×10^5^/ml (BMSCs and oral epithelium of rat fetus) were co-cultured in proximity to each other with 2:1 proportion for 14 days in DMEM (containing 15% FCS, 1% ATB and 10ng/ml insulin growth factor (IGF)). After wards they were incubated at 37˚C and 5% CO_2_. As the control group, BMSCs were used alone. An E300, Eclipse, Nikon inverted microscope (made in Japan) was used for cytological study.

### Quantitive RT-PCR

Quantitive RT-PCR was performed to assess *DMP1* and *Pax9* gene expression using RBC mRNA purificant kit (Metabion, Germany). RNA was reverse transcribed into cDNA by means of RTGO (Metabion, Germany). Glyceraldehyde 3-phosphate dehydrogenase (*GAPDH*) and *DMP1* genes were used as positive and negative control respectively. Following primers were used for RT-PCR:

- DMP1 forward primer: 5´-CCCGCAGAACCTGAAGATG- 3´
- DMP1 reverse primer: 5´-GACCCGGCAAAACAGGTAG- 3´
- Fragments: 1060bp
- Pax9 forward primer: 5´-GCCCACGTTGCTGCTTAGATTGAAA- 3´
- Pax9 reverse primer: 5´-CTCCCTCCCTTCCCGGCTCT- 3´
- Fragment: 240bp
- GAPDH forward primer: 5´-TGATGACATCAAGAAGGTGGTGAAG- 3´
- GAPDH reverse primer: 5´-TCCTTGGAGGCCATGTGGGCCAT- 3´
- Fragment: 240bp

After computing the annealing temperature according to METABION co. instruction, these genes were polymerized using the RT co. kit and results were analyzed after Electrophoresis and taking photographs.

### Ethical considerations

The number of included animals, their nutrition and maintenance status were supervised by a specialist veterinarian. The bone marrow donors were aware of the usage of their tissue samples in this study and signed the informed consent. The present study was approved by the Ethical Committee of Dental Faculty, Shahid Beheshti Medical Sciences University.

## Results

Cultured bone marrow cells expressed CD13, CD90, CD105, CD166, -34FITC and -45FITC which indicates they are mesenchymal stem cells and not hematopoietic cells (Fig 2).

Cultured embryonic epithelial cells showed positive reaction with CD104, CD120a, CD143, and CD164 which indicates the presence of odontogenic epithelium (Fig 2).

**Fig 3:**
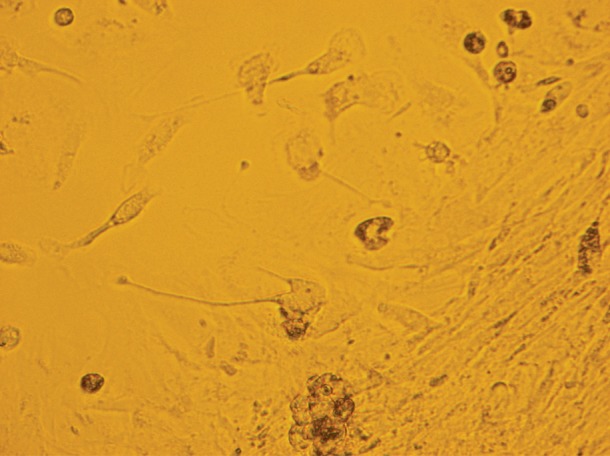
2816×2112- Aggregation of polygonal epithelial cells with round to oval nuclei. Mesenchymal cells with centrally located pale staining nuclei located near epithelial cells.

Aggregation of polygonal cells with round to oval nuclei, granular cytoplasm and desmosome junctions were observed. Mesenchymal cells with centrally located pale staining nuclei located near polygonal cells (Fig 3).

**Fig 4:**
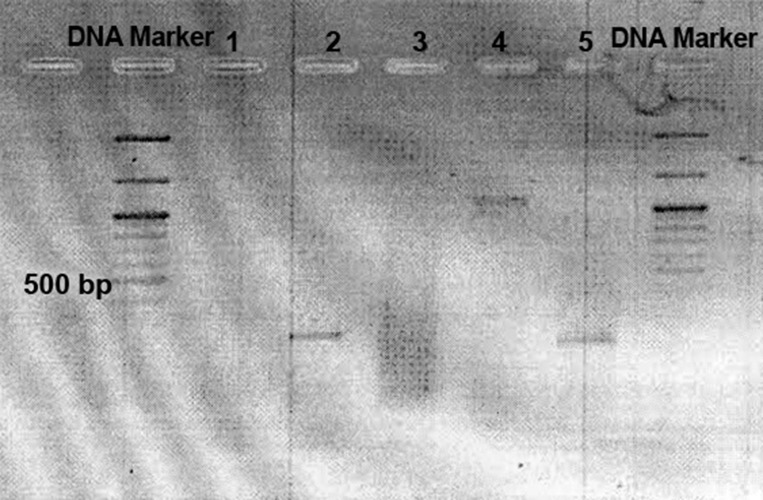
Inverted electrophoresis picture. 1. BMSCs did not express DMP1 gene when they were not in proximity to oral epithelium (as negative control), 2. Pax9 expression by BMSCs after proximity, 3. Extracted mRNA, 4. DMP1 expression of BMSCs after proximity, and 5. *GAPDH* expression by BMSCs without proximity (as positive control).

Using RT-PCR, both *DMP1* and *Pax9* genes were expressed by HBMSCs after 14 days proximity with rat odontogenic epithelium. According to the control group (BMSCs alone) RT-PCR, the result was negative (Fig 4).

## Discussion

It is well known that the interaction between inner enamel epithelium and dental papilla mesenchymal cells leads to odontoblastic and ameloblastic differentiation during tooth development. These specialized cells secrete specific mineralized materials (enamel and dentin). In the present study we replaced the epithelial cells derived from primary mouth of the rat with human BMSCs to investigate the odontogenic potential of human BMSCs. Expression of two odontogenic genes (*Pax9* and *DMP1*) by human BMSCs, indicates odontogenic capacity of these cells. Similar studies used different animals including SD rat (26, 28), 1-CR rat and SD rat (34) as mesenchymal or epithelial cell sources. We used SD rat with respect to maintenance cost and animal size. Because the first histological signs of the rat tooth development as first Branchial arch thickening become evident at day 11, this study was designed based on 11 days old rat fetuses (4).

We seeded mesenchymal and epithelial cells near to a supposition line in a liquid medium without full interconnection. This method was used due to its simplicity and speed. Previous studies have used different methods. In some studies epithelial cells were centrifuged to get cell aggregation and cells were then seeded in a semi-solid medium near mesenchymal cells (28, 35). Other methods such as transferring mesenchymal cells into a translucent membrane and adding epithelial pieces after centrifuging mesenchymal cells (19), using collagen scaffold and nano hydroxyl apatite (36), using poliglicolic acid (PGA) and poly coglycolic copolymer (PLGA) scaffolds (2, 37), silk fibron (1, 38, 39) and using a mixture of mesenchymal and epithelial cells without attention to their integration (34), were also performed.

Odontogenic potential of odontogenic and non-odontogenic stem cells has been reported in the literature (14, 15, 17, 25-27). Ohazama et al. reported that among the three kinds of non-odontogenin mesenchymal stem cells (neural stem cells, BMSCs and Embryonic stem cells), BMSCs had the best odontogenic results (26). Nakatsuka and colleagues reported differentiation of odontogenic stem cells into mature dental structures such as dentin, pulp, cementum and PDL, but could not differentiate into ameloblast (11). However, two other studies have demonstrated BMSC differentiation into ameloblast-like cells (28, 29). These cells have different origin from dental follicle stem cells (which are more differentiated): BMSCs originate from mesoderm whereas the origin of dental follicle stem cells is the neural crest (24). Comparing with DPSCs and SCAPs (two odontogenic stem cells), BMSCs have different multipotentiality profile. Some studies have reported a greater tendency to odontogenic/ osteogenic differentiation in BMSCs (40, 41), while others suggest that DPSCs and SCAPs have more tendency to differentiate into odontogenic/osteogenic lineages (41-45).

We used the BMSCs due to its good results as well as its availability and being more accessible compared with odontogenic stem cells. Because of delayed expression of *DMP1*compared with *Pax9* we used *DMP1* as a negative control knowing that loss of *DMP1* expression leads to loss of *Pax9* expression. We used *GAPDH* gene as positive control since it is expressed in all cell types and can be used as an index to measure the expression level of other genes by comparing the intensity of electrophoresis bands. This makes the results semiquantitative and more accurate.

Expression of different genes have been investigated by different authors as odontogenic differentiation indicators including Amelogenin, Ameloblastin, dentin phosphoprotein (DPP), dentin sialoprotein (*DSP*) (28), *Pax9* and DMP (25) and Lhx7 and Msx1 (26, 37). Zhang et al. reported that in the absence of *Pax9* tooth development will stop at bud stage (5). According to Li et al. *DSPP* expression indicates odontogenic differentiation and *DMP1* is expressed in the time interval between *Pax9* and DMPP expression (25). In this study we demonstrated that proximity of human BMSCs with rat epithelium leads to induction of *Pax9* and *DMP1* by BMSCs. This finding is consistent with the finding of Li et al. who reported *Pax9*, *DMP1* and *DSPP* expression by rat BMSCs (25).

## Conclusion

Expression of *Pax9* and *DMP1* by human BMSCs in the proximity of rat odontogenic epithelium in liquid medium is suggestive of odontogenic potential of these cells. Although this is the early stage of using stem cells for the production of tooth structures, it has the potential of natural tooth becoming the replacement of the lost teeth.

More studies on the HBMSCs are recommended to establish odontoblast cells with the ability to secrete dentin matrix and develop the ectodermal component of the tooth bud to produce ameloblasts and enamel.
